# The Unique Roles of Executive Function in Co‐Occurring Psychopathologies in Autism

**DOI:** 10.1002/aur.70296

**Published:** 2026-06-19

**Authors:** Shing‐Him Ng, Jet Leung, Keith Chun Lok Lee, Angela Man Wai Lam, Isaac Ho Wai Wong, Sze‐Mai Ng, Maciej Gabrysiak, Petrina Yau Pok Lau, Sandra Sau Man Chan, Tyler M. Moore, Kosha Ruparel, Raquel E. Gur, Ruben C. Gur, Oscar Wing Ho Wong

**Affiliations:** ^1^ Faculty of Medicine The Chinese University of Hong Kong Hong Kong China; ^2^ Department of Psychology The Chinese University of Hong Kong Hong Kong China; ^3^ Department of Psychiatry The Chinese University of Hong Kong Hong Kong China; ^4^ The D.H. Chen Foundation Hub of Advanced Technology for Child Health (HATCH) The Chinese University of Hong Kong Hong Kong China; ^5^ The Gerald Choa Neuroscience Institute The Chinese University of Hong Kong Hong Kong China; ^6^ Neurodevelopment and Psychosis Section, Department of Psychiatry, Perelman School of Medicine University of Pennsylvania Philadelphia Pennsylvania USA; ^7^ Children's Hospital of Philadelphia and Penn Medicine Lifespan Brain Institute (LiBI) Philadelphia Pennsylvania USA

**Keywords:** ADHD, anxiety, autism, cognition, executive function

## Abstract

Children with autism spectrum disorder (ASD) often experience co‐occurring psychiatric conditions, such as anxiety and attention‐deficit/hyperactivity disorder (ADHD), which significantly impair daily functioning. However, the underlying mechanisms linking these conditions to core ASD features remain poorly understood. This study examined relationships between anxiety, ADHD symptoms, and multi‐domain neurocognitive abilities in a clinic‐based sample of 701 Chinese children with ASD (mean age 8 years, 13.4% female), using the Hong Kong version of the Penn Computerized Neurocognitive Battery. Initial correlations revealed negative associations between cognitive performance and ADHD symptoms, but no direct links with anxiety. Ordinal regression demonstrated that poorer executive function (EF) independently predicted greater ADHD severity along a continuum from no ADHD to subclinical features to co‐occurring ADHD, in a dose‐dependent manner, even after controlling for core autism symptoms, other cognitive domains, and full‐scale IQ. Moderation analyses showed that stronger EF specifically buffered the associations between sensory hyper‐responsiveness/sensory‐seeking and anxiety symptoms—an effect not seen with other cognitive domains or full‐scale IQ. These findings indicate that EF plays a unique, compensatory role in modulating the two most common co‐occurring psychopathologies in ASD. Systematic assessment of EF could support early risk stratification, while EF‐targeted interventions may help mitigate the burden of ADHD and anxiety in children with ASD.

## 1 Introduction

Despite sharing core clinical features of autism, children with autism spectrum disorder (ASD) exhibit highly variable functional outcomes (Waizbard‐Bartov and Miller 2023). Understanding the determinants of this variability has significant clinical implications for early identification of high‐risk children with unfavorable outcomes and for the development of targeted interventions. Co‐occurring psychopathologies represent one of the most influential factors, interacting with core autism symptoms to substantially impair social, academic, and daily functioning. Attention deficit/hyperactivity disorder (ADHD) and anxiety disorders are the most prevalent co‐occurring psychiatric disorders in ASD, often emerging in early childhood (Lai et al. 2019). These conditions are associated with increased risk of subsequent psychiatric difficulties, directly impacting long‐term prognosis and quality of life (Flouri et al. 2015). However, the mechanisms underlying the high rates of co‐occurring psychopathology in ASD remain poorly understood. Genetic studies show that many variants conferring risk for ASD exert pleiotropic effects on co‐occurring psychiatric disorders (Ghirardi et al. 2021). As these genetic variants are implicated in brain functions such as neuronal activities and corticogenesis (Grove et al. 2019), neurocognitive processes represent potential common pathways. Cognitive functioning varies across individuals with ASD (Iuculano et al. 2020) and predicts adaptive functioning longitudinally (Kenny et al. 2019). Therefore, it may serve as a key pathway linking genetic risk to co‐occurring psychopathologies and long‐term prognosis.

Among the cognitive domains, executive function (EF) has emerged as particularly promising for understanding shared pathobiological pathways, as EF is proposed to serve as a compensatory mechanism in neurodevelopmental disorders (Johnson 2012) by recruiting alternative neural pathways to mitigate core deficits (Kaiser et al. 2010). Prior studies have linked EF impairments to ADHD, anxiety, and depressive symptoms (Lawson et al. 2015), and generalized psychopathology (Wong et al. 2023) in ASD. Although these findings established the importance of EF, its precise role is yet to be clarified. Most studies have examined EF in isolation (Sadozai et al. 2024), leaving open whether its effects are truly specific or reflecting broader cognitive associations. Additionally, although ASD is inherently heterogeneous, the spectrum of core symptoms severity and the variety of associated psychopathologies are rarely examined together, despite evidence that they influence one another. For example, core symptoms of ASD are associated with and may even predict both ADHD and anxiety disorder (Williams et al. 2021; Zachor and Ben‐Itzchak 2019), yet how cognitive abilities moderate these relationships remains unclear. Collectively, these gaps highlight the need for a comprehensively phenotyped cohort to disentangle the complex relationships and elucidate cognitive mechanisms underlying co‐occurring psychopathologies and identify actionable clinical targets.

The present study addresses these gaps using the Hub of Advanced Technology for Child Health (HATCH; https://www.hatchhk.org) cohort, a large, clinically characterized sample of children with ASD in Hong Kong. We examine the role of EF in relation to anxiety and ADHD symptoms, while controlling for other cognitive domains, medication status, and core symptom severity. This study aims to clarify the interrelationships between cognitive function and psychopathological outcomes in ASD in order to inform prognostic models and guide the development of targeted interventions for pediatric practice.

## 2 Methods

### 2.1 Study Population

Pre‐pubertal Chinese children (aged 6–12 years) with ASD were recruited from a university‐affiliated tertiary child and adolescent psychiatry specialist clinic to establish this Hong Kong HATCH cohort (Su et al. 2024). Diagnosis of ASD was made by child psychiatrists according to the *Diagnostic and Statistical Manual of Mental Disorders*, Fifth Edition (DSM‐5) criteria (American Psychiatric Association 2013). All diagnoses were established through comprehensive clinical assessment based on detailed developmental and clinical history obtained from the primary caregiver and direct behavioral observation of the child. Children with profound autism, psychotic spectrum disorders, or major neurological disorders (e.g., epilepsy, traumatic brain injury, and cerebral palsy) were excluded. Parents or legal guardians provided written informed consent. The study protocol followed the Declaration of Helsinki and was approved by the Joint Chinese University of Hong Kong‐New Territories East Cluster Clinical Research Ethics Committee (reference: 2021.550).

### 2.2 Clinical Profiling

Sociodemographic and clinical data were obtained from parent‐reported questionnaires and medical records. Given the high prevalence of co‐occurrence, ADHD was systematically evaluated by the attending psychiatrist for every child diagnosed with ASD at the psychiatric specialty clinics. The ADHD diagnosis was made according to the DSM‐5 criteria through a clinical interview with the primary caregiver, direct behavioral observation, and school‐based information using the SWAN questionnaire (Lai et al. 2013). This evaluation stratified participating children with ASD into three groups: autism without ADHD (no ADHD), autism with subclinical ADHD (with ADHD features), and autism with co‐occurring ADHD diagnosis (with ADHD). Prescription of psychiatric medications (medication for ADHD or antipsychotics) was recorded.

Core autism symptoms were assessed using the Chinese version of the Social Responsiveness Scale, Second Edition (SRS‐2), yielding subscales for social communication impairments (SCI) and restricted, repetitive behaviors (RRB) (Gau et al. 2013). Sensory symptoms were measured with the Sensory Experiences Questionnaire Version 2.1 (SEQ), comprising subscales for hyper‐responsiveness, hypo‐responsiveness, and sensory seeking behaviors (Baranek et al. 2006). Anxiety was quantified using the Anxiety Scale for Children—Autism Spectrum Disorder (ASC‐ASD) (Rodgers et al. 2016). In addition to reviewing the medical records, ADHD symptoms were further evaluated dimensionally using the Attention Problems subscale of the Child Behavior Checklist (CBCL) for Age 6–18 (Leung et al. 2006).

### 2.3 Cognitive and IQ Assessment

Cognitive performance was assessed using the Hong Kong Chinese version of the Penn Computerized Neurocognitive Battery (CNB‐HK) (Wong, Lam, et al. 2025), a validated instrument developed by the Brain Behavior Laboratory at the University of Pennsylvania (Gur et al. 2010; Moore et al. 2015), comprising 12 tasks evaluating four cognitive domains: social cognition (emotion recognition, emotion intensity differentiation, and age differentiation), episodic memory (words, faces, and shapes), complex cognition (set‐shifting, spatial processing, and non‐verbal reasoning), and EF (attention, working memory, and inhibition)—see Table S1 for details of the tasks. The battery was administered by trained research staff and in a quiet office, following a standardized manual. Proctors assigned validity scores for each task based on data completeness and performance quality. Invalid data (e.g., due to lack of effort or technical issues) were discarded following established quality assurance procedures. Participants with a prescription for stimulants were asked not to take them on the day of testing where possible, and medication use was recorded if taken. Tasks were scored for accuracy and speed, which were z‐transformed (z‐speed multiplied by −1 so that lower scores reflect slower performance) using local age‐standardized norms established from 412 sex‐balanced typically developing children from the community. The z‐transformed speed and accuracy scores were then summed as an efficiency score. Domain factor scores were derived using confirmatory factor analysis (CFA) on the efficiency scores (for details on quality assurance procedures, data processing, and CNB‐HK validation, see Wong, Lam, et al. 2025). To account for intellectual differences between individuals, a full‐scale IQ (FSIQ) was estimated using the short form of the Hong Kong Wechsler Intelligence Scale for Children, Fourth Edition [WISC‐IV (HK) Short Form] (Wechsler 2010).

### 2.4 Statistical Analysis

Statistical analyses were performed using R (version 4.5.2) and Jamovi (version 2.6.44). Descriptive statistics including demographics and clinical characteristics were compared across groups with *χ* ^2^ tests for categorical data and one‐way ANOVA for continuous data. Neurocognitive data missing at the task level were handled with maximum likelihood estimation for CFA, whereas missing data for the clinical rating scales and FSIQ were handled with multiple imputation using the R package ‘mice’ (*m* = 5 imputations). A correlation matrix was computed to examine the relationships within and between clinical symptoms and cognitive performance to guide subsequent analyses. Finally, moderation analysis and multivariate regression analysis were performed to examine the relationship between cognitive performance and anxiety symptoms (ASC‐ASD total score), dimensional ADHD symptoms (Attention Problem Subscale of CBCL), and categorical diagnosis (ADHD features and ADHD). Statistical significance was set at *p* < 0.05 (two‐tailed), and *p* values were adjusted for multiple testing using the Benjamini‐Hochberg false discovery rate (FDR) procedure for exploratory analysis.

## 3 Results

### 3.1 Study Population Characteristics

A total of 701 pre‐pubertal Chinese children with autism were recruited and completed the study (mean age = 8.03 years; 13.4% female; mean FSIQ = 97.6, SD = 16.2). Participants were stratified by co‐occurring ADHD status: 325 with no ADHD, 48 with ADHD features, and 328 with co‐occurring ADHD. This clinician‐based classification aligned with parent‐rated ADHD symptoms on the CBCL Attention Problems subscale, which showed progressively higher scores across the no ADHD, ADHD features, and ADHD groups (*p* < 0.001; *ηp* ^2^ = 0.16). Age and FSIQ differed significantly across ASD groups (both *p* < 0.001; *ηp* ^2^_Age_ = 0.084; *ηp* ^2^_FSIQ_ = 0.023), with age lower in the ADHD features group and IQ lower in both ADHD groups compared to the no ADHD group, whereas sex distribution (*p* = 0.087) and parental education were comparable (*p =* 0.437). Core autism features, including social communication and interaction deficits, restricted/repetitive behaviors (as measured by SRS‐2), and sensory symptoms (as measured by SEQ), were significantly more severe in the ADHD features and ADHD groups compared to the no ADHD group (all *p* < 0.05). In contrast, anxiety symptoms (ASC‐ASD total score) were similar across groups (*p* = 0.498). Children in the ADHD features and ADHD diagnosis groups were more likely than those in the no ADHD group to be prescribed ADHD medications (2.1% and 62.2%, respectively, vs. 0%; *p* < 0.001) and antipsychotics (4.2% and 14.3% respectively, vs. 3.1%; *p* < 0.001). Demographic and clinical characteristics of the study population, stratified by ADHD status, are summarized in Table 1.

**TABLE 1:** Demographics and clinical characteristics of participants with autism stratified into ADHD groups.

	Missing data	Without ADHD		With ADHD features		With ADHD		Statistics			
	%	Mean/*n*	SD/%	Mean/*n*	SD/%	Mean/*n*	SD/%	*F*/*χ* ^2^	df	*p*	ηp2/Cramer's V
*n*		325	46.36	48	6.85	328	46.79				
Age	—	7.94	1.73	7.2	1.63	8.24	1.74	8.54	2689	< 0.001***	0.024
Sex	—										
Male		290	89.23	38	79.17	279	85.06	4.89	2	0.087	0.084
Female		35	10.77	10	20.83	49	14.94				
Highest parental education attained	0.71										
Primary or less		3	0.93	0	0.00	2	0.61	5.88	6	0.437	0.065
Secondary		107	33.23	13	27.66	125	38.23				
Tertiary		212	65.84	34	72.34	200	61.16				
Prescribed medications	—										
ADHD medications		0	0.00	1	2.08	204	62.20	323	2	< 0.001***	0.679
Antipsychotics		10	3.08	2	4.17	47	14.33	24.8	2	< 0.001***	0.206
Taking stimulant during CNB		0	0	0	0	104	31.7	—			
Social Responsiveness Scale‐2	1										
SCI subscale		62.3	20.8	66.5	17.90	70.4	18.80	13.80	2, 691	< 0.001***	0.038
RRB subscale		11.2	6.8	12.1	6.37	14.4	6.74	17.6	2, 691	< 0.001***	0.048
Total score		73.5	26.8	78.6	23.20	84.8	24.60	15.6	2, 691	< 0.001***	0.043
Sensory experiences questionnaire	1										
Hyporesponsiveness		1.78	0.60	1.79	0.61	1.93	0.60	5.34	2, 691	0.005**	0.015
Hyperresponsiveness		2.15	0.56	2.19	0.49	2.26	0.56	3.06	2, 691	0.047*	0.009
Sensory seeking		2.03	0.67	2.04	0.61	2.2	0.68	5.14	2, 691	0.006**	0.015
Child Behavior Checklist	1										
Attentional problems subscale		58	8.55	61.6	9.97	66.4	10.30	63.4	2, 691	< 0.001***	0.155
Normal		268	83.75	34	72.34	183	55.96	61.7	4	< 0.001***	0.211
Borderline range		15	4.69	2	4.26	26	7.95				
Clinical range		37	11.56	11	23.40	118	36.09				
ASC‐ASD total score	1	17.8	9.81	18	9.88	18.8	11.40	0.72	2, 691	0.498	0.002
FSIQ	6.6	100.0	17.60	95.3	16.90	95.3	14.40	7.80	2, 652	< 0.001***	0.023

*Note:* *p < 0.05, **p < 0.01, ***p < 0.001.

Abbreviations: ADHD: attention deficit and hyperactivity disorder; ASC‐ASD: anxiety scale for children—ASD.

The cognitive performance on the CNB‐HK was compared across the three autism groups and the non‐autistic normative children whose data were used to establish z‐score norms. ANCOVA, controlling for age and sex, revealed significant group differences across all four cognitive domains (all *p* < 0.001), with a general pattern of progressively poorer performance from the no ADHD group to the ADHD features group, and to the co‐occurring ADHD group. Post hoc comparisons showed that all three autism groups performed significantly (*p*_Tukey_ < 0.05), or at trend level (*p*_Tukey_ = 0.073 for Social Cognition of ADHD features group), worse than the non‐autistic normative sample on social cognition and episodic memory. In contrast, deficits in EF and complex cognition were observed only in the co‐occurring ADHD group relative to the non‐autistic group (*p*_Tukey_ < 0.001); the no ADHD and ADHD features groups did not differ significantly from non‐autistic children on these two domains (*p*_Tukey_ = 0.182–0.597) (Table 2).

**TABLE 2:** Comparisons of CNB‐HK cognitive domain efficiency scores across ASD subgroups and non‐autistic normative sample.

Cognitive domain	Non‐autistic		ASD						Statistics			
			Without ADHD		With ADHD features		Co‐occurring ADHD					
	Mean	SD	Mean	SD	Mean	SD	Mean	SD	*F*	df	*p*	*ηp* ^2^
Social cognition	0.22	0.72	−0.04	0.90	−0.17	0.96	−0.19	0.94	10.77	3, 1095	< 0.001	0.029
Episodic memory	0.19	0.69	−0.02	0.83	−0.24	0.89	−0.16	0.89	10.61	3, 1095	< 0.001	0.028
Complex cognition	0.13	0.68	0.07	0.91	−0.10	0.90	−0.16	0.88	8.32	3, 1095	< 0.001	0.022
Executive function	0.12	0.69	0.10	0.90	−0.08	0.71	−0.20	0.90	8.54	3, 1095	< 0.001	0.034

Post hoc comparisons with non‐autistic normative sample												
	Social cognition			Episodic memory			Complex cognition			Executive function		
	Mean difference	*t*	*p*_Tukey_	Mean difference	*t*	*p*_Tukey_	Mean difference	*t*	*p*_Tukey_	Mean difference	*t*	*p*_Tukey_
Without ADHD	−0.2117	−3.139	0.009**	−0.1938	−3.037	0.013*	−0.0817	−1.248	0.597	−0.0955	−1.466	0.458
With ADHD features	−0.3197	−2.425	0.073	−0.4077	−3.268	0.006**	−0.2256	−1.762	0.292	−0.2571	−2.019	0.182
Co‐occurring ADHD	−0.3678	−5.575	< 0.001***	−0.3281	−5.258	< 0.001***	−0.3038	−4.744	< 0.001***	−0.3729	−5.853	< 0.001***

*Note:* Results were controlled for age and sex; *p < 0.05, **p < 0.01, ***p < 0.001.

Abbreviation: ASD: autism spectrum disorder.

### 3.2 Intercorrelations Among Core Symptoms, Co‐Occurring Psychopathology and Cognition

**FIGURE 1:**
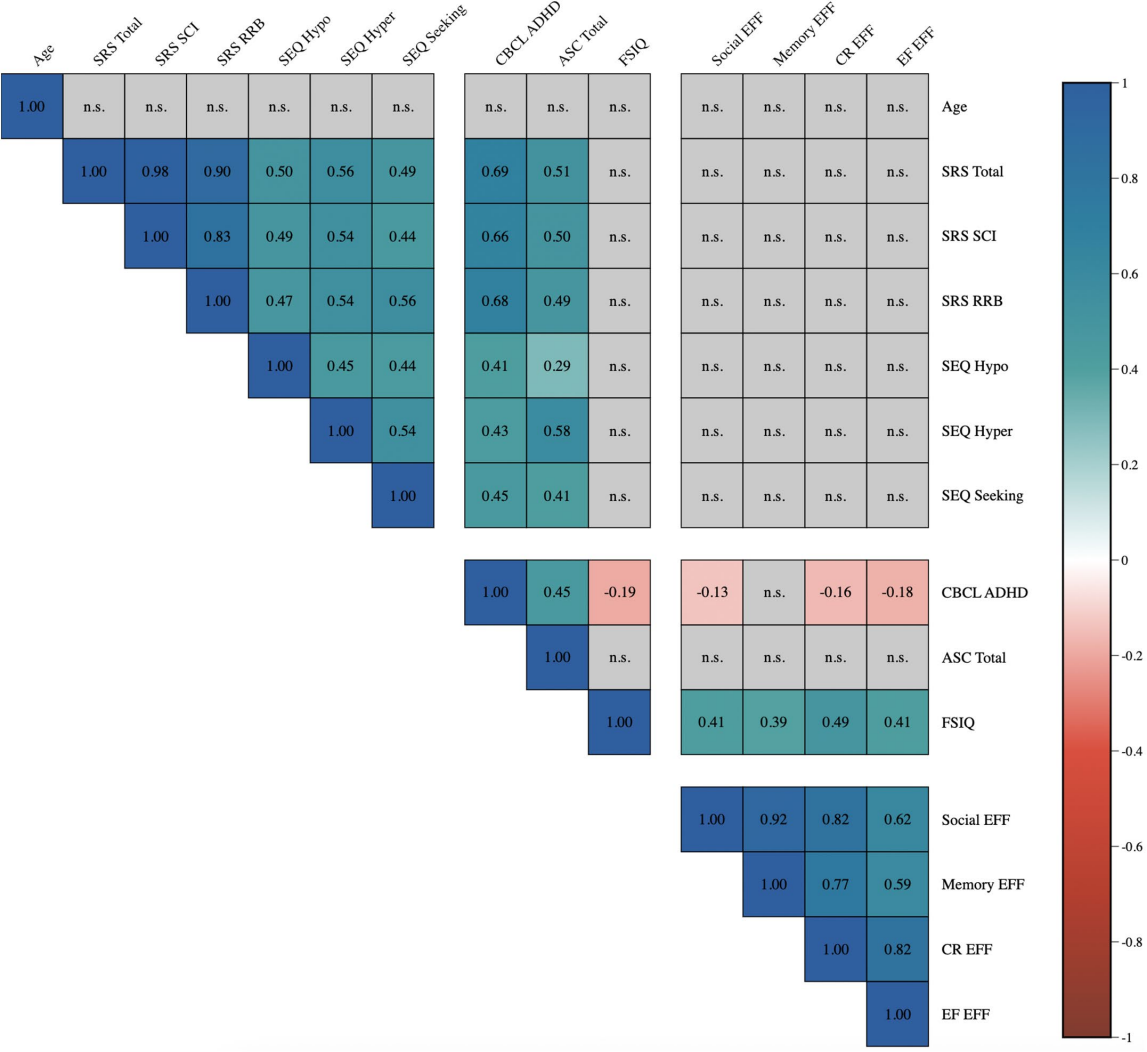
Spearman correlation matrix of clinical symptoms and cognitive domain efficiency scores in children with ASD. Heatmap displaying Spearman's rho correlations of core autism features (SRS‐2 Total SCI and RRB subscales), sensory symptoms (SEQ hyporesponsiveness, hyperresponsiveness, and sensory seeking), ADHD Symptoms (CBCL ADHD), Anxiety (ASC‐ASD total), FSIQ, and cognitive domain efficiency scores (social cognition, episodic memory, complex cognition, and executive function). Non‐significant correlations are not displayed.

Bivariate correlations among core autism symptoms, psychopathological outcomes, and cognitive domain efficiency scores are presented in Figure 1. Core autism symptoms (Subscales from SRS‐2 and SEQ) showed moderate to strong positive associations with both anxiety (ASC‐ASD total score, *r* = 0.29–0.58, all *p* < 0.001) and ADHD symptoms (CBCL Attention Problem Subscale score, *r* = 0.41–0.68, all *p* < 0.001). Sensory hyperresponsiveness (SEQ hyperresponsiveness subscale) was the strongest correlate of anxiety (*r* = 0.58), while SRS total score was the strongest correlate of ADHD symptoms (*r* = 0.68). Cognitive domain efficiency scores of the CNB‐HK were weakly to moderately negatively associated with ADHD symptoms (*r* = −0.13 to −0.18, all *p* < 0.001), with EF efficiency score showing the strongest correlation (*r* = −0.18). Their associations with anxiety were near zero and non‐significant (*r* = −0.03 to 0.01, *p* = 0.368–0.803). FSIQ associated positively with all the cognitive domain efficiency scores (*r* = 0.39 to 0.49, *p* < 0.001), was negatively associated with ADHD symptoms (*r* = −0.19, *p* < 0.001) but unrelated to anxiety (*r* = −0.02, *p* = 0.673).

### 3.3 Executive Function as a Predictor of Clinical Diagnosis ADHD but Not Parent‐Reported Symptoms

For parent‐reported dimensional ADHD symptoms (CBCL Attention Problems subscale score), multivariate linear regression revealed that EF was not a significant predictor (*B* = −0.75, SE = 0.55, *p* = 0.17) after controlling for age, sex, FSIQ, stimulant use during CNB‐HK, performance in other cognitive domains, and core autism symptom severity (SRS‐2 total score). In contrast, SRS‐2 total score emerged as a strong positive predictor of CBCL Attention Problems subscale scores (*B* = 0.26, SE = 0.01, *p* < 0.001) and accounted for the majority of variance in the model (adjusted *R* ^2^ = 0.509). For clinician‐classified ADHD status (no ADHD, ADHD features, or co‐occurring ADHD), ordinal logistic regression showed a significant negative stepwise association with EF (*B* = −0.53, OR = 0.59, 95% CI [0.43–0.82], *p* = 0.002). No other cognitive domain or FSIQ significantly predicted clinician‐rated ADHD status. The overall model explained 33.5% of the variance in ADHD severity category (Nagelkerke pseudo *R* ^2^ = 0.335). These findings indicate that EF efficiency uniquely predicts clinician‐diagnosed ADHD status in a dose‐dependent manner, independent of core autism symptom severity. In contrast, the core autistic symptom severity accounted for the majority of the variance of parent‐reported ADHD symptoms (Table 3).

**TABLE 3:** Regression models predicting ADHD symptoms and diagnosis with cognitive domains.

Predictor	Estimate	SE	Statistic	*p*	OR (95% CI)
Linear regression to predict CBCL attention problems subscale score					
Intercept	45.08	2.77	16.29	< 0.001***	—
Executive function	−0.75	0.55	−1.37	0.170	
Complex cognition	−1.05	0.78	−1.34	0.180	
Social cognition	0.58	0.90	0.65	0.518	
Episodic memory	−0.02	0.82	−0.03	0.980	
SRS‐2 total score	0.26	0.01	23.42	< 0.001***	
Age	0.18	0.17	1.06	0.291	
Sex (male)	−1.57	0.82	−1.91	0.056	
Full‐scale IQ (FSIQ)	−0.04	0.02	−2.16	0.031*	
Taking simulant during CNB‐HK	3.21	0.80	4.02	< 0.001***	
Ordinal regression to predict clinician‐classified ADHD status					
Executive function	−0.53	0.17	−3.12	0.002**	0.59 (0.43–0.82)
Complex cognition	0.25	0.24	1.01	0.312	1.28 (0.80–2.05)
Social cognition	−0.10	0.27	−0.37	0.715	0.90 (0.53–1.55)
Episodic memory	0.02	0.25	0.07	0.941	1.02 (0.62–1.67)
SRS‐2 total score	0.01	0.00	4.10	< 0.001***	1.01 (1.01–1.02)
Age	0.04	0.05	0.78	0.434	1.04 (0.94–1.14)
Sex (male)	−0.44	0.24	−1.88	0.061	0.64 (0.40–1.02)
Full‐scale IQ (FSIQ)	−0.01	0.01	−1.47	0.142	0.99 (0.98–1.00)

*Note:* *p < 0.05, **p < 0.01, ***p < 0.001. Estimates were pooled across five imputed datasets using Rubin's rules. Linear regression: *R* ^2^ = 0.515, adjusted *R* ^2^ = 0.509. Ordinal regression: Nagelkerke pseudo *R* ^2^ = 0.335. Usage of stimulants during CNB‐HK was removed from the ordinal model due to complete separation.

Abbreviations: CBCL: Child Behavior Checklist; CNB: Penn's computerized neurocognitive battery; SRS‐2: Social Responsiveness Scale‐2.

### 3.4 Executive Function as a Moderator of Sensory Feature Links to Anxiety

Although direct associations between cognitive domain performance and anxiety symptoms were not observed, EF is theorized to play a compensatory role in mitigating the impact of core autism features. We therefore examined whether EF moderated the well‐established associations between the core autism features and anxiety symptoms (Williams et al. 2021), which were also observed in the correlational patterns in our data (Figure 1).

**FIGURE 2:**
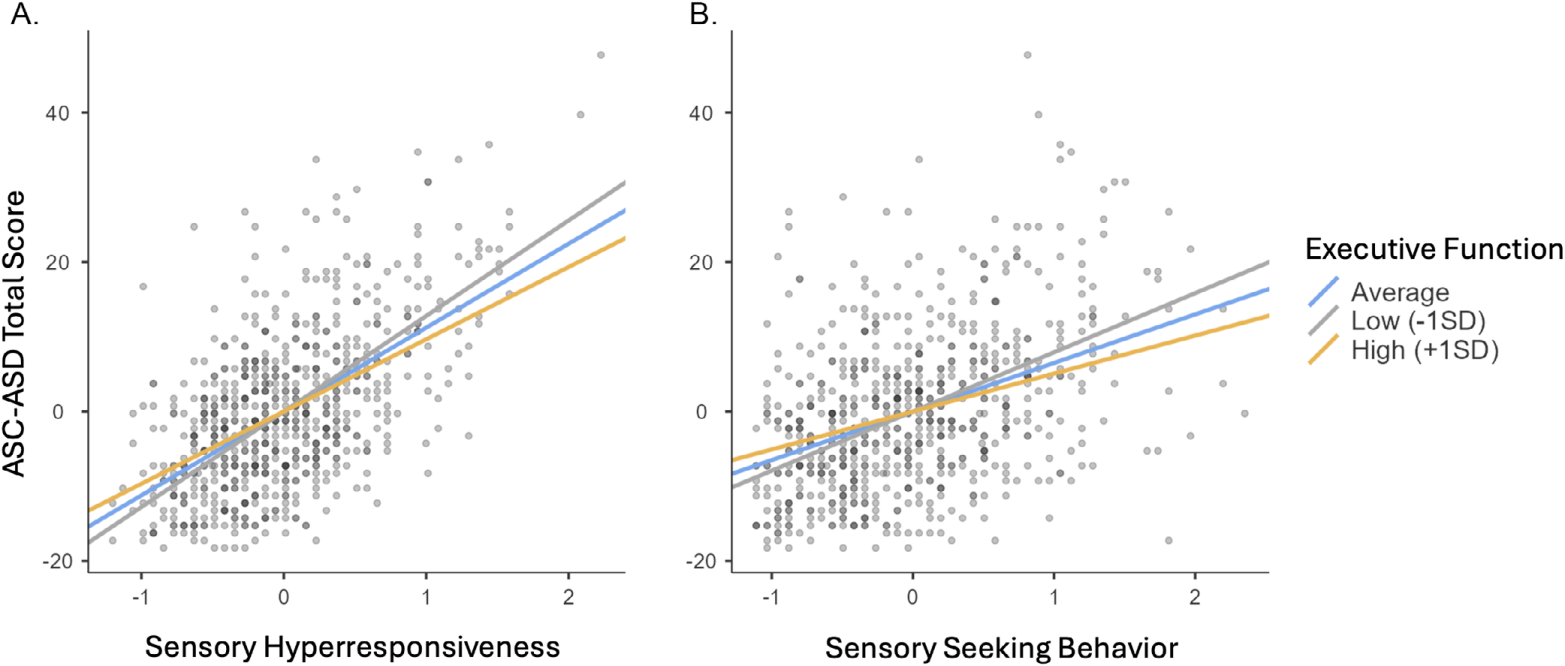
Executive function moderates the association between sensory and anxiety symptoms in children with ASD. Interaction plots ([A] Sensory hyperresponsiveness and [B] sensory seeking behavior) illustrating the moderating effect of executive function (EF) on the relationship between sensory and anxiety (ASC‐ASD total score) symptoms, with EF probed at low (−1 SD), mean, and high (+1 SD) levels.

Multiple linear regression models tested moderation by EF on the relationships between five core autism features (SCI and RRB subscales from SRS‐2; hyporesponsiveness, hyperresponsiveness, and sensory seeking subscales scores from SEQ) and anxiety symptoms (ASC‐ASD total score), controlled for age, sex, FSIQ, efficiency scores of the other cognitive domain, and stimulant use during CNB‐HK. After FDR correction, EF significantly moderated the associations of sensory hyperresponsiveness (*B* = −1.61, standardized *β* = −0.08, Δ*R* ^2^ = 0.005, *p* = 0.016, *p*_adj_ = 0.047) and sensory seeking (*B* = −1.43, standardized *β* = −0.08, Δ*R* ^2^ = 0.007, *p* = 0.019, *p*_adj_ = 0.047) with anxiety symptoms. Moderation effects for hyporesponsiveness, SRS‐2 RRB, and SRS‐2 SCI did not survive FDR correction but had a trend towards statistical significance (*p*_adj_ = 0.061–0.064) (Table 4). Simple slopes analyses revealed a consistent buffering pattern: the positive associations between sensory hyperresponsiveness/sensory‐seeking and anxiety were significantly stronger at lower levels of EF efficiency (−1 SD) than at higher levels (+1 SD). Specifically, the low‐versus‐high EF contrast was significant for sensory hyperresponsiveness (estimate = 2.83, SE = 1.21, *p* = 0.019) and seeking (estimate = 2.44, SE = 1.09, *p* = 0.026) (Figure 2). Thus, better EF weakens the link between heightened sensory symptoms and anxiety, potentially reflecting compensatory effect. In contrast, no significant moderation effects were observed for the other cognitive domains on the relationships between core autism features and anxiety symptoms. A nominally significant interaction emerged for complex cognition moderating the association between sensory hyperresponsiveness and anxiety, but this did not survive correction for multiple comparisons (B = −1.33, SE = 0.67, *t* = −1.99, *p* = 0.047, *p*_adj_ = 0.234, Table S2).

**TABLE 4:** Moderation effect by executive function on the associations between core autism features and anxiety symptoms.

Predictor	Moderator	*B*	*β*	SE	*t*	*p*	*p*_adj_
Sensory hyperresponsiveness	Executive function	−1.609	−0.076	0.664	−2.426	0.016*	0.047*
Sensory‐seeking behavior		−1.429	−0.082	0.606	−2.359	0.019*	0.047*
Sensory hyporesponsiveness		−1.547	−0.079	0.738	−2.095	0.037*	0.061
Restricted, repetitive behavior		−0.103	−0.061	0.052	−1.976	0.049*	0.061
Social communication impairment		−0.034	−0.058	0.019	−1.854	0.064	0.064

*Note:* *p < 0.05. Results were controlled for age, sex, usage of ADHD medication during CNB, FSIQ, and efficiency scores of other cognitive domains. Benjamini‐Hochberg correction was applied for multiple comparisons. Pooled estimates across five imputed datasets using Rubin's rules. No. of imputations = number of datasets contributing valid estimates.

## 4 Discussion

In this study, we systematically profiled clinical symptoms, multidomain cognitive functioning, and their interactions among school‐aged children with ASD. We clarified the specific role of EF in two common co‐occurring psychiatric conditions—anxiety and ADHD. Key strengths of this study include its relatively large, well‐characterized sample of 701 children with ASD, in which ADHD status was determined through systematic clinical evaluation and cognitive performance was rigorously standardized against normative data from non‐autistic children. Statistical analyses were also stringently controlled for demographics, medication effect, other cognitive domains, and general intelligence. These methodological designs and sample size provide a robust framework for evaluating neurocognitive effects across multiple domains, detecting modest effects reliably, and interpreting non‐significant results with greater confidence. Poorer EF independently predicted more severe clinician‐rated ADHD in a dose‐dependent manner. EF also significantly moderated the associations between sensory hyperresponsiveness and sensory seeking with anxiety symptoms. These associations with ADHD severity and the moderating effects on anxiety were specific to EF and were not observed for other cognitive domains. Together, these findings indicate that EF plays a unique role in shaping psychiatric outcomes in children with ASD.

However, the interpretation of our findings should also be considered within the context of our methodological approach in assessing EF. It is well established that performance‐based measures of EF (such as the CNB‐HK) and informant‐rated measures (such as the Behavior Rating Inventory of Executive Function [BRIEF]) show only modest correlations and appear to capture partially distinct constructs (Toplak et al. 2013). Performance‐based tasks assess cognitive processes under structured testing conditions, whereas rating scales reflect abilities in everyday environments. In the present study, we focused on performance‐based EF to allow direct comparison with other cognitive domains within the same validated neurocognitive battery. While this approach offers advantages of objectivity and cross‐domain comparability, it is important to acknowledge that performance‐based measures may not fully capture the functional executive difficulties experienced by autistic children in daily life (Kenworthy et al. 2008). This choice of a performance‐based assessment may therefore have influenced the pattern of associations observed in this study. Nevertheless, the CNB‐HK EF tasks have demonstrated associations with activation of frontoparietal executive networks in functional neuroimaging studies, providing support for their construct validity (Roalf et al. 2014; Satterthwaite et al. 2013). A second methodological consideration concerns our operationalization of EF. Although cognitive flexibility is widely regarded as a core component of EF (Friedman and Miyake 2017), the set‐shifting task (Penn Conditional Exclusion Test) loaded onto the Complex Cognition domain rather than EF in the factor analyses of both the original CNB (Moore et al. 2015) and the CNB‐HK (Wong, Lam, et al. 2025). Consequently, the latent EF factor in the present study primarily reflects sustained attention, working memory, and inhibitory control. This definition of EF should be taken into account when comparing our findings with studies that conceptualize EF more broadly to include cognitive flexibility.

### 4.1 Dose‐Dependent Link Between Executive Function and Co‐Occurring ADHD in ASD

Executive dysfunction, particularly deficits in sustained attention and inhibitory control, is a well‐established causal cognitive underpinning of ADHD (Schachar 2023). It is therefore expected that EF directly predicts the co‐occurrence of ADHD among children with ASD. Our study extends the clinical understanding of interactions between EF and co‐occurring ADHD in ASD by examining the intermediate group with subclinical features, which are not uncommon and can affect adaptive functioning (Yerys et al. 2019). By classifying the study population along a spectrum of ADHD symptoms—ranging from none to subclinical features to a full ADHD diagnosis—we utilized ordinal logistic regression to demonstrate that poorer EF is associated with ADHD severity in a dose‐dependent manner. EF demonstrated a moderate protective effect (OR = 0.59 per 1 SD increase), such that for every standard deviation increase in EF efficiency, the odds of being classified in a higher ADHD severity category decreased by 41%. These findings suggest that the relatively preserved EF skills in the subclinical ADHD group may mitigate the impact of ADHD symptoms, thereby preventing disorder‐level functional impairment. However, increasing the sample size in this intermediate subgroup would strengthen the reliability of this finding. Furthermore, EF remained a significant predictor of ADHD status even after controlling for SRS‐2 total score, performance in other cognitive domains, and FSIQ. This indicates that the relationship between EF and ADHD in autism is specific and not merely a reflection of general cognitive ability or confounded by overall severity of core autism symptoms.

However, the lack of a predictive association between EF and parent‐rated ADHD symptoms (CBCL Attention Problems subscale) contrasts with the significant dose‐dependent relationship observed with clinician‐classified ADHD status. Several factors may contribute to this discrepancy. One possible explanation relates to the ecological validity of performance‐based EF measures (Toplak et al. 2013), which may not fully capture the everyday behavioral manifestations that parents observe and report on the CBCL. Additionally, substantial behavioral symptom overlap exists between ASD and ADHD, which can reduce the discriminant validity of parent‐report measures. For example, social indifference or repetitive behaviors characteristic of ASD may be misinterpreted by parents as inattention or hyperactivity (Antshel and Russo 2019; Krakowski et al. 2020). In our data, CBCL Attention Problems scores were strongly correlated with SRS‐2 total score (*r* = 0.68, *p* < 0.001), and up to 15.56% of children without a clinician diagnosis of ADHD scored in the borderline or clinical range on the CBCL. These findings are consistent with previous reports that parent‐rated CBCL Attention Problem scores demonstrate only modest discriminant validity for identifying clinical ADHD diagnoses among children with ASD (Rau et al. 2020).

In contrast, clinician‐rated ADHD status in this study incorporated multi‐source information, including parent reports, direct behavioral observation, and teacher's ratings, to provide a more comprehensive and reliable clinical identification of ADHD in ASD. This approach aligns with recommendations that clinical interviews and multi‐informant assessments should supplement parent‐rated screening tools when evaluating ADHD in children with ASD (Yerys et al. 2016). By integrating multiple sources of information, clinician‐assessed ADHD status offers a more valid estimate of EF's role in ADHD symptomatology than parent‐reported CBCL scores. The robust dose‐dependent relationship between EF and clinician‐classified ADHD severity observed in this study supports EF as a meaningful target for intervention in children with co‐occurring ASD and ADHD.

### 4.2 Executive Function as a Buffer for Core Autism Features Contributing to Anxiety

Anxiety is the second most common co‐occurring psychiatric condition in autism, with multiple contributing factors identified, including core autism symptoms (Williams et al. 2021), demographics (Dubin et al. 2015), gastrointestinal symptoms (Wong, Ng, et al. 2025; Wong et al. 2021), and cognition. Although this study relied on parent‐reported measures for anxiety assessment, prior research has demonstrated that such reports show good agreement with clinical consensus diagnoses (Storch et al. 2012). While anxiety symptoms can also overlap with core autism features—such as reduced social engagement arising from social anxiety or inherent social indifference—the ASC‐ASD used in this study was specifically designed to capture anxiety presentations unique to autistic populations (Rodgers et al. 2016). This ensures that the anxiety symptoms assessed in the current study are distinct from autism‐related behaviors, thereby reducing potential measurement confounds.

Unlike the relatively consistent association between EF and ADHD, the relationship between executive dysfunction and anxiety in ASD has been more mixed in the literature. While some studies report significant associations between EF difficulties and anxiety (Dieckhaus et al. 2021), others have found no relationship (Gardiner and Iarocci 2018; Solomon et al. 2008). In contrast, cognitive flexibility, a component that was not captured within our EF factor, has shown more consistent cross‐sectional and longitudinal associations with internalizing symptoms in the autistic populations (Lei et al. 2022).

In the present study, EF (which captured components of sustained attention, working memory, and inhibition) did not show a direct association with anxiety symptoms. Instead, EF appears to function as a moderator, buffering the impact of core autism features on anxiety. Notably, EF significantly moderated the associations between sensory hyperresponsiveness (standardized *β* = −0.08, Δ*R* ^2^ = 0.005) and sensory seeking (standardized *β* = −0.08, Δ*R* ^2^ = 0.007) with anxiety symptoms. Although these moderation effects were small in magnitude, they remained significant after controlling for other cognitive domains, FSIQ, and core autism symptoms and survived FDR correction. These findings suggest that even modest improvements in EF may help buffer the impact of sensory symptoms on anxiety in children with ASD. In contrast, other cognitive domains did not demonstrate a comparable moderating effect. These findings highlight the unique role of EF in mitigating anxiety symptoms in children with ASD.

Sensory atypicalities, particularly hyperresponsiveness, are hypothesized to contribute to anxiety in autistic children by creating a sense of diminished control over an unpredictable environment (Russo et al. 2025), and exaggerated responses to environmental or situational stimuli (Green and Ben‐Sasson 2010). Attentional control, a core component of EF, may mitigate these effects by enabling flexible redirection and disengagement from anxiety‐provoking stimuli (Shi et al. 2019). Stronger EF, particularly better executive control, may therefore reduce or buffer anxiety symptoms in autistic children through compensatory mechanisms. Conversely, anxiety itself may impair central executive efficiency (Eysenck et al. 2007), potentially creating a self‐perpetuating cycle that further undermines this compensatory capacity. This bidirectional relationship underscores the importance of addressing EF deficits in interventions targeting anxiety in ASD.

Currently, no pharmacological treatment (e.g., antidepressant) has demonstrated robust efficacy in managing significant anxiety symptoms in autistic children (Gupta and Gupta 2024). Evidence from the general population suggests that working memory training may reduce anxiety symptoms (Wang et al. 2023); however, it remains unclear whether similar benefits extend to autistic children. Future research should investigate the potential of EF‐targeted behavioral interventions, such as working memory training or attentional control strategies, to alleviate both ADHD and anxiety symptoms in this population. Such efforts could provide critical insights into the development of tailored, non‐pharmacological treatments aimed at improving co‐occurring psychiatric symptoms in ASD.

### 4.3 Clinical Implications and Future Research Directions

EF has been reported to be impaired at the group level in ASD (Demetriou et al. 2018), and other neurodevelopmental disorders (Sadozai et al. 2024). However, given the heterogeneous clinical outcomes in ASD, the variable rates of co‐occurring psychiatric conditions, and their putative associations with EF, executive dysfunction is unlikely to be a universal phenomenon at the individual level. As highlighted in a recent review, the specific ways in which children with ASD or ADHD exhibit impaired EF remains uncertain, as many studies fail to account for symptom co‐occurrence across these disorders (Kofler et al. 2024). In the present study, children with ASD but without ADHD demonstrated EF performance comparable to non‐autistic peers, underscoring the variable occurrence of executive dysfunction in ASD rather than its ubiquity. Given that the defining symptoms of ADHD are directly tied to executive dysfunction (Schachar 2023), and the high prevalence (up to one‐third) of co‐occurring ADHD in ASD (Lai et al. 2019), future studies on EF in ASD must control for the confounding influence of ADHD symptoms to avoid misattribution of deficits. This variability also highlights the importance of examining intermediate groups with subclinical ADHD features. Future research should investigate whether EF dysfunction directly drives ADHD symptom severity or, conversely, whether relatively preserved EF protects against progression to a full ADHD diagnosis despite presence of comparable symptom levels. The high co‐occurrence of ADHD in ASD, combined with shared cognitive pathways (e.g., EF as a common underpinning) and overlapping genetic variants between the two conditions (Mattheisen et al. 2022), raises important questions about diagnostic boundaries. Specifically, future biological and pathophysiological studies could explore whether co‐occurring ADHD represents a distinct entity or whether it should be reconceptualized as a dimensional profile within the autism spectrum. Such investigations could provide critical insights into the shared and distinct mechanisms underlying these conditions, ultimately informing diagnostic frameworks and intervention strategies.

Clinically, our findings suggest that EF assessment may serve as a valuable prognostic tool in ASD. Systematic evaluation of EF could help identify individuals at heightened risk for co‐occurring psychopathology, enabling early, targeted interventions and personalized treatment planning. From a therapeutic standpoint, while current interventions for ASD primarily address core social and behavioral features, EF emerges as a promising target for improving functional outcomes. Emerging evidence indicates that EF in autistic children can be enhanced through targeted cognitive training programs (Cavalli et al. 2022). Additionally, neuromodulation techniques, such as transcranial magnetic stimulation (TMS) (Ameis et al. 2020), are actively being explored as novel treatment modalities to improve EF in ASD.

## 5 Limitations

Despite the relatively large sample size and the use of comprehensive methods, several limitations warrant consideration. First, the cross‐sectional design of this study limits the ability to draw causal inferences. Bidirectional or multidirectional interactions likely exist between psychopathology, cognitive function, and core autism symptoms. Longitudinal studies are needed to clarify these complex relationships and the potential compensatory role of EF in ASD. Second, anxiety symptoms were assessed solely through parent‐reported questionnaires, without clinical evaluation to identify specific anxiety disorders. This limits the granularity of our understanding of anxiety presentations in this population. Future studies should incorporate clinician‐administered diagnostic tools to provide a more nuanced characterization of anxiety symptoms in autistic children. Third, the study included only ethnically Chinese children and excluded individuals with profound autism, which restricts the generalizability of the findings. Expanding future research to include these populations is important to ensure that findings are representative of the autism spectrum. Additionally, autistic girls were underrepresented in the current cohort, which limits our ability to examine potential sex‐specific roles of EF in co‐occurring psychopathology in ASD. Fourth, EF was examined as a unitary construct. Due to the factor structure of the CNB‐HK, cognitive flexibility was not included in the EF factor. Consequently, the EF construct examined in this study primarily reflects sustained attention, working memory, and inhibitory control. It remains unclear whether the observed associations with psychopathology are driven by specific EF subcomponents or by global EF abilities. Furthermore, as a performance‐based battery, the CNB‐HK may not fully capture the complex, integrative, and context‐dependent nature of executive functioning in everyday environments. Future research would benefit from incorporating both performance‐based measures and rating scales to provide a more comprehensive assessment of EF in ASD.

## 6 Conclusion

The present study highlights the unique role of EF in modulating common co‐occurring psychopathology in children with ASD, a pattern not observed in other cognitive domains. Specifically, poorer EF predicted ADHD clinical severity in a dose‐dependent manner; executive dysfunction may drive the progression from subclinical symptoms to clinically significant impairment. Conversely, EF demonstrated a buffering effect, attenuating the association between sensory symptoms and anxiety symptoms. These findings underscore the compensatory potential of EF and its critical roles in shaping clinical outcomes in ASD. However, the variability of EF in autism further emphasizes the need for nuanced research that accounts for co‐occurring conditions and symptom heterogeneity. Future longitudinal studies are also needed to clarify causal relationships and identify the underlying biological mechanisms driving these interactions. Clinically, EF emerges as a promising prognostic marker and treatment target with the potential to improve functional outcomes in individuals with ASD. Systematic EF assessment in autism may serve as a valuable tool for identifying individuals at heightened risk for co‐occurring psychopathology, enabling early intervention and personalized treatment planning. Overall, EF represents a critical domain for advancing both research and clinical practice in ASD. By incorporating EF evaluation into standard clinical practice and targeting executive abilities through evidence‐based interventions, clinicians can better address the complex needs of children with ASD and ultimately improve their long‐term developmental and functional outcomes.

## Author Contributions

S‐.H.N., J.L., K.C.L.L., A.M.W.L., I.H.W.W., S‐.M.N., and O.W.H.W. were involved in the conceptualization and design of the study. S‐.H.N., J.L., K.C.L.L., A.M.W.L., and O.W.H.W. were involved in data acquisition. S‐.H.N., J.L., K.C.L.L., A.M.W.L., M.G., and O.W.H.W. were involved in data processing and data analysis. S‐.H.N., J.L., K.C.L.L., A.M.W.L., P.Y.P.L., and O.W.H.W. were involved in writing and editing the manuscript. S.S.M.C., T.M.M., K.R., R.E.G., and R.C.G. oversaw the manuscript writing process. All authors read and approved the final manuscript.

## Funding

This research was supported by The D.H. Chen Foundation.

## Ethics Statement

All procedures performed in studies involving human participants were in accordance with the ethical standards of the institutional and/or national research committee and with the 1964 Helsinki Declaration and its later amendments or comparable ethical standards. The study was approved by the Joint CUHK‐NTEC CREC (Ref: 2021.554).

## Consent

Written informed consent was obtained from the parents or legal guardians of the participants.

## Conflicts of Interest

The authors declare no conflicts of interest.

## Supporting information


**Table S1:** lists the individual tasks included in the Hong Kong version of the Penn Computerized Neurocognitive Battery (CNB‐HK) and their respective cognitive domains.
**Table S2:** presents the results of the moderation analyses examining the effects of Social Cognition, Episodic Memory, and Complex Cognition on the associations between core autism features and anxiety symptoms.

## Data Availability

O.W.H.W. has access to all the data in the study and takes responsibility for the integrity of the data and the accuracy of the data analysis. The datasets generated during and/or analyzed during the current study are available from the corresponding author on reasonable request.
