# Supplementary material for: The Unique Roles of Executive Function in Co‐Occurring Psychopathologies in Autism
Source: Autism Res. 2026 Jun 19;19(8):e70296. doi: 10.1002/aur.70296 (PMC13471759; doi:10.1002/aur.70296)
Supplement: Supplementary file 1 [file AUR-19-0-s001.docx]

| **Supplementary Table S1.** | | |
| --- | --- | --- |
| The Tasks and their Respective Cognitive Domain in the Hong Kong version of the Penn Computerized Neurocognitive Battery | | |
| Neurocognitive domain | Cognitive function | Task |
| Episodic Memory | Visual Memory | Visual Object Learning Test |
|  | Word Memory | Word Memory Task |
|  | Face Memory | Face Memory Task |
| Social Cognition | Emotion Intensity Differentiation | Emotion Differentiation Test |
|  | Emotion identification | Emotion Identification Test |
|  | Age Differentiation | Age Differentiation Test |
| Complex Cognition | Non-verbal reasoning | Matrix Reasoning Test |
|  | Set-shifting | Penn Conditional Exclusion Test |
|  | Spatial Processing | Line Orientation Test |
| Executive Function | Attention | Penn Continuous Performance Test |
|  | Working Memory | Letter N-Back Task |
|  | Inhibition | Go/No-Go Task |
|  | | |

| **Supplementary Table S2** | | | | | | |  |
| --- | --- | --- | --- | --- | --- | --- | --- |
| Moderation Effect by Social Cognition, Episodic Memory and Complex Cognition on the Associations between Core Autism Features and Anxiety Symptoms | | | | | | |  |
| Predictor | Moderator | B | SE | t | *p* | *p_adj_* |  |
| Social Communication Impairment | Social Cognition | 0.016 | 0.020 | 0.801 | 0.423 | 0.516 |  |
| Restricted, Repetitive Behavior |  | 0.084 | 0.057 | 1.470 | 0.141 | 0.448 |  |
| Sensory Hyporesponsiveness |  | -0.443 | 0.682 | -0.650 | 0.516 | 0.516 |  |
| Sensory Hyperresponsiveness |  | -0.860 | 0.640 | -1.340 | 0.179 | 0.448 |  |
| Sensory-Seeking Behavoir |  | -0.605 | 0.606 | -0.998 | 0.319 | 0.516 |  |
| Social Communication Impairment | Episodic Memory | 0.021 | 0.021 | 0.989 | 0.323 | 0.404 |  |
| Restricted, Repetitive Behavior |  | 0.093 | 0.063 | 1.490 | 0.136 | 0.404 |  |
| Sensory Hyporesponsiveness |  | 0.428 | 0.778 | 0.551 | 0.582 | 0.582 |  |
| Sensory Hyperresponsiveness |  | -0.930 | 0.710 | -1.310 | 0.191 | 0.404 |  |
| Sensory-Seeking Behavoir |  | -0.692 | 0.676 | -1.020 | 0.306 | 0.404 |  |
| Social Communication Impairment | Complex Cognition | -0.003 | 0.020 | -0.176 | 0.860 | 0.959 |  |
| Restricted, Repetitive Behavior |  | -0.003 | 0.057 | -0.052 | 0.959 | 0.959 |  |
| Sensory Hyporesponsiveness |  | -0.535 | 0.712 | -0.751 | 0.453 | 0.755 |  |
| Sensory Hyperresponsiveness |  | -1.330 | 0.665 | -1.990 | 0.047^*^ | 0.234 |  |
| Sensory-Seeking Behavoir |  | -0.779 | 0.625 | -1.250 | 0.213 | 0.533 |  |
| *Notes. * p < 0.05. Results were controlled for age, sex, FSIQ and efficiency scores of other cognitive domains Benjamini-Hochberg correction was applied for multiple comparisons. Pooled estimates across 5 imputed datasets using Rubin's rules. No. of imputations = number of datasets contributing valid estimates.* | | | | | | |  |
|  |  |  |  |  |  |  |  |
